# Microplastics exposure: implications for human fertility, pregnancy and child health

**DOI:** 10.3389/fendo.2023.1330396

**Published:** 2024-01-04

**Authors:** Rewa E. Zurub, Yusmaris Cariaco, Michael G. Wade, Shannon A. Bainbridge

**Affiliations:** ^1^ Interdisciplinary School of Health Sciences, Faculty of Health Sciences, University of Ottawa, Ottawa, ON, Canada; ^2^ Environmental Health Science and Research Bureau, Health Canada, Ottawa, ON, Canada; ^3^ Department of Cellular and Molecular Medicine, Faculty of Medicine, University of Ottawa, Ottawa, ON, Canada

**Keywords:** plastics pollution, microplastic, pregnancy, placenta, DOHAD

## Abstract

Plastics found in our everyday environment are becoming an increasing concern for individual and population-level health, and the extent of exposure and potential toxic effects of these contaminants on numerous human organ systems are becoming clear. Microplastics (MPs), tiny plastic particles, appear to have many of the same biological effects as their plastic precursors and have the compounded effect of potential accumulation in different organs. Recently, microplastic accumulation was observed in the human placenta, raising important questions related to the biological effects of these contaminants on the health of pregnancies and offspring. These concerns are particularly heightened considering the developmental origins of health and disease (DOHaD) framework, which postulates that *in utero* exposure can programme the lifelong health of the offspring. The current review examines the state of knowledge on this topic and highlights important avenues for future investigation.

## 1 Plastic pollution

Plastics are synthetic or semi-synthetic polymers, developed after the 19^th^-century Industrial Revolution. Due to their many useful characteristics, including being lightweight, infinitely mouldable, having low production cost, broad chemical resistance, and ease to manufacture and transport (1), they are widely used in food packaging (i.e., containers, plastic bags), building products (i.e., pipes, vinyl cladding), electronics, and transportation materials (1). The development of plastics has also revolutionized medicine with life-saving devices and the availability of sterile, single-use instruments and personal protective equipment. However, the excessive use of plastics has led to a throw-away culture resulting in increasing amounts of environmental plastic pollution that resists degradation.

Plastic pollution is an accumulation of synthetic plastic products in the environment, disrupting the habitats and health of wildlife and humans. The rapidly rising output of disposable plastic goods is currently exceeding our capacity to handle its disposal, leading to the emergence of plastic pollution as one of the most urgent environmental issues (2). As previously reviewed by Hirt and colleagues (3), plastic waste reached 359 million metric tons in 2018 (3), with estimates that between 4.8 and 12.7 million metric tons are reaching the ocean each year, contributing to 80% of the plastic pollution in the world’s oceans and seas (3). Plastic trash is also carried to sea by major rivers, which acts to distribute waste, picking up more and more garbage as it moves downstream. When plastic trash gets caught up in an ocean’s current, it can be transported around the world.

Many single use plastic products have a functional lifespan of minutes to hours, yet they may persist in the environment for hundreds of years. Plastic degradation is a very slow process, with fragmentation and degradation of plastic polymers occurring by physical forces, ultraviolet (UV) rays, temperature changes and biodegradation in the environment. The resulting breakdown products are smaller plastic fragments, known as micro and nano -plastics (4)

## 2 Microplastics and nanoplastics

Microplastics (MPs) are generated by the breakdown of larger plastic products. Microplastics are omnipresent in our environment, being found in large quantities in oceans, rivers, ground water, sediments and soil environments, sewage, and even the air we breathe (5). Most plastics in use have a strong resistance to biodegradation (6). However, they are susceptible to mechanical and photochemical processes that can break them down into micro and nanoscale particles (6). Nanoplastics (NPs) are plastic particles ranging in size from 1nm – 1μm (7). MPs and NPs demonstrate similar characteristics and biological effects; however, NPs demonstrate higher biological mobility and bioavailability because of their small size, which enables them to pass through biological membranes relatively easily (8). For the purposes of the current review, the term micro-nano-plastics (MNP) will be used to describe all plastic fragments < 5 mm – and, as such, will include both MPs and NPs.

**Figure 1:**
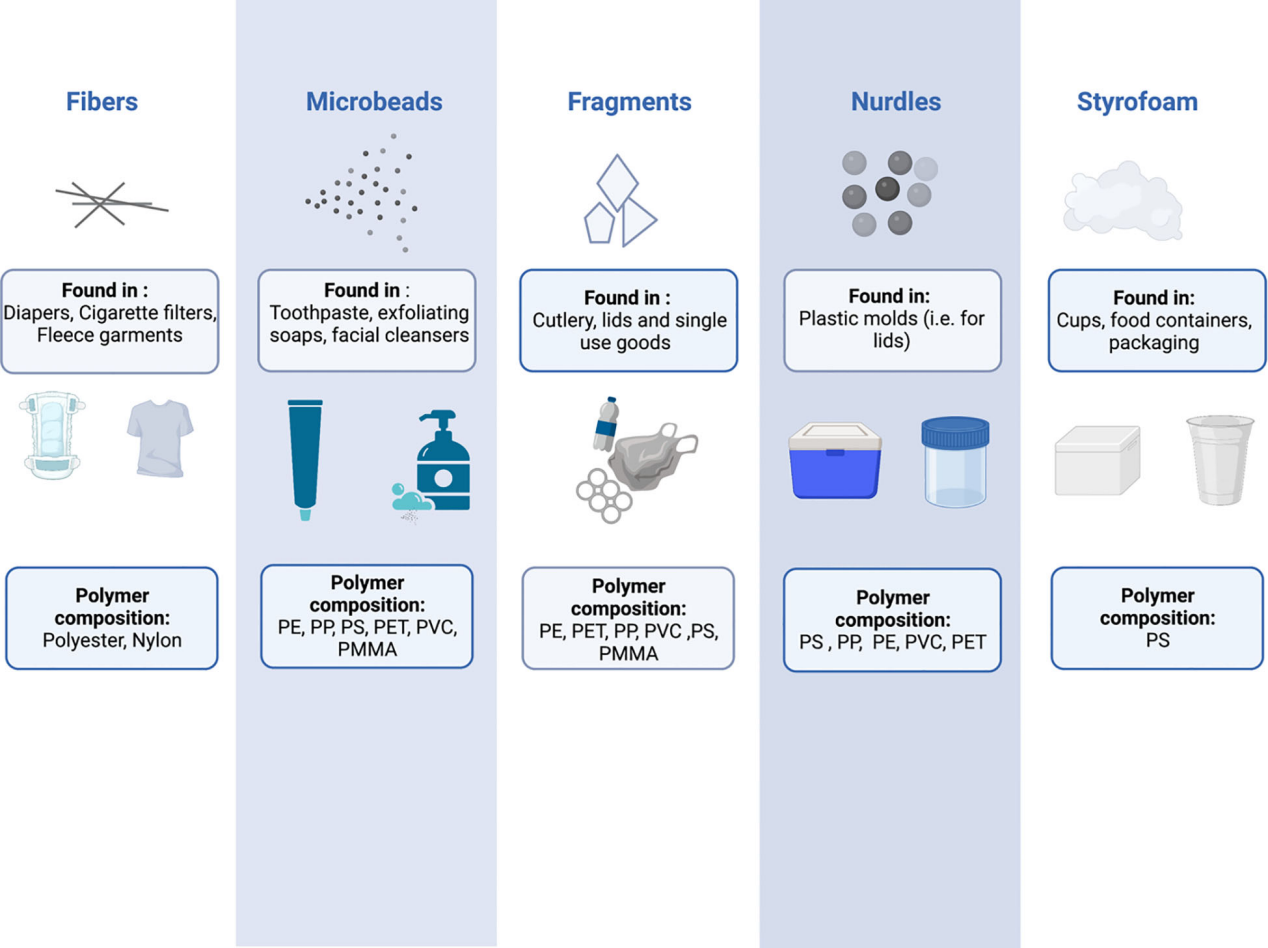
The shapes, compositions, and potential sources of common micro and nanoplastics. PE, Polyethylene; PP, Polypropylene; PS, Polystyrene; PET, Polyethylene terephthalate; PVC, Polyvinyl chloride; PMMA, Polymethyl methacrylate.

MNPs can be further characterized by their polymer composition and shape – characteristics that are intimately linked to the plastic product source from which they were derived (**Figure 1**). Plastics are made up of various polymers, including polyethylene (PE), polypropylene (PP), polystyrene (PS), polyvinyl chloride (PVC), polyethylene terephthalate (PET), polycarbonate (PC), polymethacrylate (PMMA), and polyurethane (PU) (3). However, polyethylene, polypropylene and polystyrene are the three most common occurring polymers (5), being found in a countless number of household and personal care products (9, 10), cosmetic products (11), toothpaste (10, 12, 13) and plastic food containers (14). The shape of MNPs is also varied, and includes fibres, microbeads, fragments, nurdles and Styrofoam (3). The types and sources of plastic pollution have been reviewed in detail elsewhere (15–21).

It is important to note that MNPs are not just pure plastic polymers, rather they are associated with a diverse mixture of organic molecules and/or metals. Commercial plastics contain many additives that can leech out of plastic into the surrounding environment or tissue(s), as they are not covalently linked to the polymer matrix. A recent review estimated that over 10,000 unique chemicals are used at various stages in plastics manufacturing, of which roughly 2,400 have been identified as chemicals of regulatory concern (22). Further, the hydrophobic surface of MNPs can absorb environmental contaminants, particularly polyaromatic hydrocarbons (23). There is concern that chemicals contained within MNPs, or those absorbed to their surface, can be carried into the human body and released into various tissue beds (24). In this way, MNPs act as a vehicle for toxic exposure to a number of xenobiotics, which may bypass typical physiological defences such as drug-metabolizing enzymes in the gut and liver and induce direct effects to the cells/tissues surrounding the internalized MNPs (25). Several chemicals known to leach from plastics are well known to induce a variety of adverse health conditions in humans, including to developing fetuses exposed *in utero* (26, 27). However, investigations specifically exploring the developmental toxicity of MNPs, and the many associated chemicals, are limited.

## 3 Routes of exposure and adverse human health outcomes

**Figure 2:**
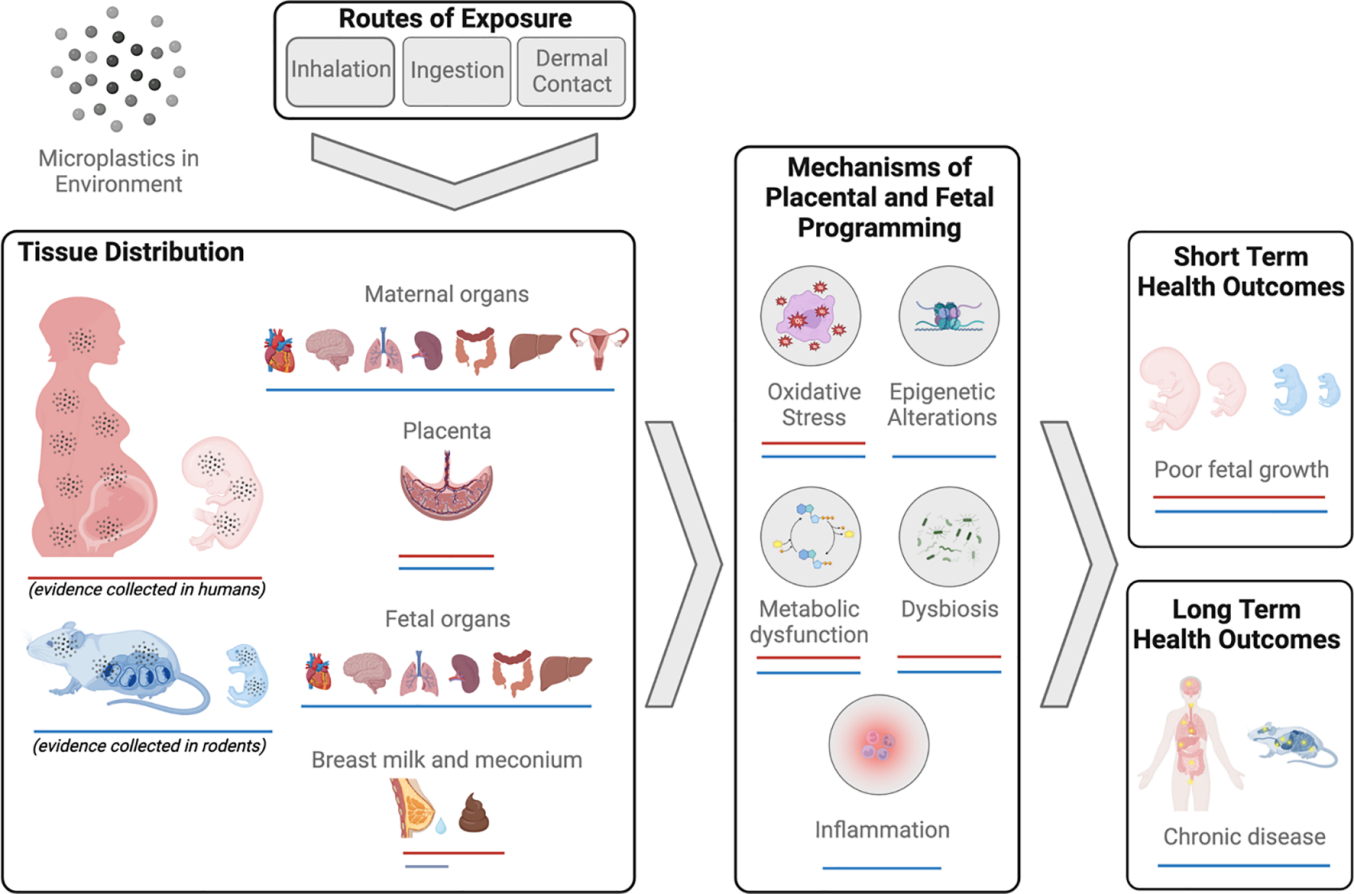
Summary of evidence of micro-nanoplastics (MNPs) exposure and impacts on reproduction and development in humans and mammals.

There are three routes through which the human body is exposed to MNPs – inhalation, ingestion and dermal contact (28) (**Figure 2**). It is estimated that an individual will be exposed to approximately 74,000-121,000 MPs per year, with ingestion and inhalation considered the primary routes of exposure (29). As this estimate does not consider NPs, it is likely that total MNP particle exposure is in fact considerably higher. Importantly, most MNPs can cross the physiological barriers of the lungs, gut, and skin. The mechanisms underlying this translocation are poorly understood and are beyond the scope of the current work, however, they have been reviewed in detail elsewhere (28).

In humans, MNPs have been found in a diverse range of biological samples, including blood (30, 31), urine (32), sputum (33), feces (34, 35), and breast milk (36, 37). Further, MNP accumulation has been identified in numerous organ systems including lung (38–43), colon and spleen (44).

Microplastics have more recently been identified in human placenta tissue (45–50) and meconium (35, 36) demonstrating direct exposure to the fetus and raising concerns for developmental toxicity and long-term health consequences for the offspring. While the scope of MNP contamination and human exposure has become widely apparent within the last decade, relatively few studies have focused explicitly on the reproductive consequences of MNP exposure, particularly in humans. The limited body of work in this area, specifically that focuses on the effects of MNP exposure on mammalian reproduction, is described below and summarized in **Figure 2**.

## 4 Effects of MNP exposure on mammalian fertility

### 4.1 Fertility effects in adult males

Several adverse reproductive effects are observed in male mammals following oral exposure to MNPs of various size and with varying duration. For example, male rodents’ oral exposure to PS-MNP leads to accumulation within the testis (51–55), coupled to disruption of the seminiferous epithelium (51, 52, 54, 56–60), evidence of localized oxidative stress and mitochondrial dysfunction (47), and over-expression of pro-inflammatory cytokines in the testis (52, 53). This same exposure is associated with disruption of the blood-testis barrier (52, 58, 59, 61), with *in vitro* studies demonstrating oxidative stress, endoplasmic reticulum stress and misfolding/degradation of tight junctional proteins in Sertoli cells (62, 63). There are clear functional consequences of these exposures, as MNP exposure in rodent models leads to reduced sperm quantity and quality (51–54, 57, 59, 61, 64, 65) in addition to reduced testicular androgen production (57) and circulating levels of testosterone (51, 54, 56, 57, 61) and luteinizing hormone (LH) (51, 54, 56, 57), suggesting that MNP exposure may have important implications in the pituitary-gonadotropin endocrine signalling pathways, testis function and sperm quality in male mammals [see D’Angelo and Meccariello for a review on this topic (66)]. It is noteworthy that an exponential rise in global plastic production (4) coincides with a well-documented, population-wide decline in human sperm production which appears to be accelerating since 2000 (67).

Male fertility, fetal health and the long-term health of offspring are dependent on the epigenetic programming events that occur during spermatogenesis, events that can be adversely disrupted by exposures to various testicular toxicants (68, 69). Epigenetic modifications play a crucial role in regulating gene expression and developmental processes, including germ cell differentiation and sperm production. While there are currently no studies to date examining the toxicant effects of MNPs on the sperm epigenome in mammals, there is strong evidence that common additives found within MNPs (i.e. phthalates and BPA) can in fact disrupt this critical developmental process. In rodent models, exposure to phthalates and BPA can induce alterations in DNA methylation patterns (70–73), histone modifications (73, 74), and non-coding RNA expression within the germline. These changes can disrupt normal epigenetic programming during critical windows of spermatogenesis, leading to impaired sperm development, reduced sperm quality, and compromised fertility (70, 73–75). Similar associations have been observed in human populations, with several studies demonstrating a correlation between urine phthalate and/or BPA metabolite concentrations and differential methylation patterns in the sperm, often in promoter regions of genes related to cellular growth and development, coupled to poor sperm quality and fertility outcomes (74, 76–79). Furthermore, the intergenerational and transgenerational effects of phthalates and BPA on germ cell epigenetic marks have been observed, indicating the potential for long-lasting impacts on future generations (70, 71).

### 4.2 Fertility effects in adult females

Like most studies investigating the reproductive impact of MNPs in males, PS-MNPs are among the most widely studied plastic particles in relation to female reproductive toxicity in mammals (80). In both a rat and mouse model, oral exposure to PS-MNPs results in the accumulation of these particles within uterine tissue (81) and in various ovarian compartments, including within growing follicles (54, 80–85). Ovaries of these exposed rodents have reduced weight, decreased expression of cytoskeletal proteins, and demonstrate altered follicle dynamics, with a reduction in the number of growing and mature follicles and increased atretic and cystic follicles (80). In parallel, distinct changes in reproductive hormone signalling are observed, with reductions in the circulating concentrations of estradiol (E_2_) and anti-mullerian hormone (AMH), and increased concentrations of LH, follicle stimulating hormone (FSH) and testosterone (54, 80, 84). Exposed rodents demonstrate functional/fecundity consequences of this MNP exposure, with measurable changes in estrous cycle duration, decreased ovarian reserve, lower embryo implantation rates and smaller litter sizes (54, 84). The mechanistic underpinning of this reproductive dysfunction is thought to be in large part driven by MNP-induced oxidative stress. Ovarian tissues of exposed rodents demonstrate markers of oxidative stress, such as malondialdehyde (MDA) (80, 81, 84, 86), with noted disturbances in total antioxidant capacity and increased evidence of apoptosis (84). Similarly, human granulosa cells (COV434) exposed to PS-MNPs *in vitro* likewise demonstrate increased evidence of lipid peroxidation, with decreased protein levels of superoxide dismutase (SOD2) and glutathione (GSH) antioxidant systems, and decreased cell viability (84). The accumulation of MNPs in female reproductive organs and the resulting oxidative stress are thought to promote excess fibroblasts proliferation and fibrosis (87–91). Furthermore, evidence of pro-inflammatory signalling is observed in these exposed tissues (81). It should be noted that very little work to date has examined the presence or toxicity of MNP exposure in human female reproductive tissues, and this should be a prioritized focus of research endeavours moving forward. However, MNPs were recently detected in follicular fluid of patients undergoing fertility treatment (92). The mean concentration of the MNPs in these samples was ~ 120 MNP/, with the most predominant polymers present being PVC, PE, PS, PP and PU, found in conjunction with several common plastic-related particles, including common pigments, solubilizers and fillers. Importantly, the authors demonstrated compromised bovine oocyte maturation when cultured in the presence of these same MNPs *in vitro*, at similar concentrations to those measured. Further, these exposed oocytes demonstrated significant proteomic alteration, with differential levels of proteins involved in oocyte function, oxidative stress, and DNA damage (92). To date, there have been no investigations examining the impact of MNPs on the oocyte epigenome in any mammalian species, however, the epigenome of female mammalian gametes is likewise known to be adversely impacted by many additive compounds found in MNPs (93, 94). As such, there is very likely additional adverse effects of these plastic particles on oocyte health, and the health of subsequent generations, driven in part by altered oocyte epigenetic imprinting. Collectively, this patchwork of findings collected across mammalian species provides compelling evidence of the toxic effects of MNPs on female reproductive health and fecundity.

## 5 MNP Exposure in pregnancy

### 5.1 Evidence of accumulation, translocation and adverse effects of MNP in the placenta

Mounting evidence suggest that MNPs accumulate within and affect the proper functioning of the placenta – the vital organ of pregnancy responsible for all maternal-fetal exchange (95).The presence of MNP accumulation in placental tissue of rats treated with PS-MNPs was first described in 2020 (96) and has since been reproduced in many studies in mice (97–99), with observed structural and functional consequences (96–101). Exposed females (MNPs between 100 nm-10 μm) have smaller placentas (96, 98), reduced numbers of glycogen-containing cells within the placental endocrine-functioning junctional zone (100), and poorly developed feto-placental vasculature (100). The remodelling of the uterine spiral arteries is also compromised, likely the result of uterine and placental immune cell imbalances (i.e., decreased uterine natural killer cells, altered macrophage ratio) (97). In addition, transcriptomic and metabolic analyses of MNP-exposed placentas demonstrate disturbed amino acid, glucose and cholesterol metabolism and complement/coagulation cascades pathways (100, 101).

More recently, MNP accumulation has been observed in human placenta tissue of otherwise healthy pregnancies delivered both vaginally and by C-section (36, 45–50, 102, 103). The number of MNPs measured varied across patients, ranging from 0.28-9.55 particles/g tissue (50), with the most common polymers identified as PE, PS, PA, PU, and PVC (36, 46, 47, 50). Grossly, MNPs were found in both the fetal and maternal compartments of the placenta, along with the chorioamniotic membranes (48). More detailed investigations identified microplastic-like particles within the syncytiotrophoblast cellular layer of the placenta, both free within the cytoplasm and encapsulated within structures located below the plasma membrane (i.e. vacuoles, lipid droplets, vesicular bodies, lysosomes, peroxisomes), as well as within the pericytes and fetal vascular endothelial cells located within the chorionic villous structures (49).

While *in vivo* functional investigations pose an ethical and logistical challenge in human populations, *in vitro* studies carried out in different human placenta cell lines demonstrate a clear potential for human placental MNP uptake and functional alterations. Using the immortalized HTR-8/sVneo extravillous cytotrophoblast cell line, PS-MNP exposure resulted in MNP accumulation within the cytoplasm, followed by increased production of reactive oxygen species (ROS), enhanced production of pro-inflammatory cytokines (e.x., TNF-α and IFN-γ), cell cycle arrest and, ultimately, reduced cellular viability (104). These cells also demonstrate altered gene expression profiles, with increased expression of genes required for regulation of leukocyte differentiation, cell cycle, apoptotic process, and cellular adhesion. Functionally, these MNP-exposed cells demonstrated impaired cell motility and invasion capacities, indicating that MNP exposure may negatively impact the invasive placentation process, required for the establishment of a robust utero-placental circulation needed to support adequate fetal development (104, 105) Studies have also been carried out in BeWo and JEG-3 cells (106, 107) – both choriocarcinoma cell lines representative of the chorionic villous cytotrophoblast and syncytiotrophoblast cell lineages, that directly facilitate maternal-fetal exchange.

A combination of *in vitro*, animal, and human *ex vivo* studies demonstrate placental cell uptake of MNPs is enhanced by smaller size and greater concentration (106–112). Observations in both maternal mice and rats exposed to PS-NPs during gestation, ranging in size from 20 to 500nm, demonstrated the presence of these particles in fetal liver (96, 108, 109), heart (96, 109), brain (99, 108, 109), lung (108, 109), and kidney (109). Interestingly, a similar exposure using PE-MPs (10-45µm) resulted in MP accumulation exclusively in fetal kidneys (110). In humans, MNPs have been measured in fetal meconium (36, 46, 47) and amniotic fluid (102), and using an *ex vivo* human placenta perfusion model, a size dependent transfer of MNPs from the maternal to fetal circulation has been described (111, 112). These exposed human placenta tissues also demonstrated dysregulated expression of genes and proteins related to inflammation and iron homeostasis (113). Collectively, these data demonstrate placental accumulation and translocation of MNPs into the fetal compartment, leading to concern that maternal MNP exposure during pregnancy may result in short- and long-term adverse health outcomes for the offspring.

### 5.2 MNP exposure during pregnancy and effects on progeny

Given the vital importance of placental health and function for fetal development, it is unsurprising that MNP exposure in pregnancy is associated with altered fetal growth profiles. Mouse models of gestational exposure to PS-MNPs, ranging in size from 90nm – 5μm, demonstrate pronounced fetal growth restriction in the last half of pregnancy (E15.5-E17), with fetal weights on average 12-15% smaller than non-exposed fetuses (98, 100, 114). The feto-placental weight ratio is also reduced (114), a finding consistent with fetal growth restriction suggesting inadequate nutrient transfer capacity to support fetal weight gain (115). These authors also observed decreased umbilical cord length in the MNP-exposed fetuses (114), a finding described in murine models of hypoxia-mediated fetal growth restriction (116, 117) and found in human cases of intra-uterine growth restriction (IUGR) and fetal distress (118). Most studies have reported no impact of maternal MNP exposure on total litter size, however, some reports of embryonic lethality and resorption have been reported in both murine and chick embryo model systems (98, 119), the latter attributed to significant embryonic malformations and developmental delays.

MNP-induced fetal growth restriction is further extended to observations of reduced birth and neonatal body weight. In both rat and mouse models, exposure to PS-MNPs (70-100 nm) during pregnancy reduced neonatal pup weight by 7-15%, in some cases in a sex-dependant fashion (96, 120–123). Specifically, two studies report reduced neonatal weights only for female offspring (121, 122). Interestingly, a paralleled decrease in placental expression of 11β-hydroxysteroid dehydrogenase (*Hsd11b1*) was uniquely observed in exposed female fetuses (121). As placental HSD11B1 enzyme is a critical regulator of fetal cortisol exposure *in utero*, the authors speculate that dysregulated glucocorticoid signalling may, in part, be responsible for the sex-specific differences in neonatal weight observed. Curiously, there is one report of altered neonatal sex ratio at birth, skewed in favor of male offspring, coupled to a reduced live birth rate in a mouse model of gestational PE-MNP exposure. However, in this model, the body weights of male and female pups were equally reduced at 6 hrs after birth (123). In human populations very little is known about the impact of maternal MNP exposure on fetal growth and offspring birthweight. However, a recent study conducted by Jeong and colleagues (45), for the first time, reported an inverse correlation between placental MNP accumulation and birthweight in IUGR pregnancies (r = - 0.82, p < 0.001). Similar relationships were observed for neonatal length at birth, head circumference and 1 minute APGAR scores. MNPs were detected in all 13 cases of IUGR examined, with up to 38 distinct MNPs measured per sample. PE and PS were the most abundant polymers identified, and the MNPs ranged in size from 2.9 to 34.5μm. While this study has a small sample size, and some methodology questions are outstanding (i.e. how much placenta tissue was examined/case)?, it certainly provides concern regarding the impact of *in utero* MNP exposure on fetal growth and development that warrants further investigation.

An increasing body of evidence suggests that *in utero* exposure to microplastics not only adversely affects fetal and neonatal body weight but also compromises fetal organ development. For example, skeletal muscle tissue collected from term murine fetuses exposed to PS-MNPs *in utero*, demonstrate significant dysplasia, with dysregulated expression of genes involved in muscle tissue development, lipid metabolism, and skin formation (100). In the post-natal period (day 14) mice exposed to MNPs *in utero* and during lactation demonstrate a substantial reduction in the number of proliferative cells within the hippocampus, with reduced numbers of neural stem cells – indicative of abnormal brain development (124). These offspring demonstrate neurophysiological and cognitive deficits in a gender-specific manner. Evidence collected using a chick embryo model likewise points to detrimental effects of *in utero* MNP exposure on nervous system development, including the observation of neural tube defects (98). Post-natal observations in both murine and chick models additionally indicate adverse effects of gestational MNP exposure on the size and histological organization of the developing liver, spleen and heart (119, 120, 123), with evidence of oxidative stress and dysregulated immune cell infiltration. Importantly, findings of altered fetal/neonate body and organ weight are shown to persist into adulthood in some cases (120, 123), emphasizing the potential for adverse short- and long-term health outcomes for offspring exposed to environmental MNPs during pregnancy.

It should be noted that not all results collected to date have likewise demonstrated reduced fetal, birth or neonatal organ weights following MNP exposure *in utero*. Rather, other groups have found no differences, or even increased rodent pup weight up to 1 week after delivery (109, 110, 124). These observed discrepancies can likely be attributed to the wide range of MNP exposure protocols and experimental methods used across studies. A large number of studies were carried out using PS-MNPs, often in the nano-particle size range (25-900 nm) ^97,99,106,111,116–119,121^, however a few used larger PS- or PE-MNPs (1-45μm) (110, 114, 119, 123) which may not demonstrate similar bioavailability and/or trans-placental transfer profiles. Lengths of maternal MNP exposure also varied some beginning exposure up to 80 days prior to mating (123) and others continuing exposure until the time of weaning (120, 124). In fact, Jeong et al. reported no changes in fetal weight profiles at gestational day 14, but increased pup weight at 7 days post-delivery – findings they attributed to postnatal MNP exposure via breastmilk (124). Further, differences in route of administration (oral vs inhalation vs injection), coupled to differences in dosing reporting practices, makes direct comparison of pregnancy outcome data from various MNP exposure models a challenge to interpret.

Infants who suffered IUGR, specifically those who experience rapid growth in early life (i.e “catch up” growth) are more susceptible to long-term effects including low stature, development of cardiovascular disease, hypertension, type 2 diabetes, metabolic syndrome and insulin resistance (125). Studies have investigated MNPs exposure in relation to metabolic and neurodevelopment impacts. Maternal exposure to PS-NPs has been associated with adverse effects on metabolic functions detected in pups post-delivery (126–131). Studies observed maternal exposure to PS-MPs (0.5– 5μm) resulted in long term metabolic adverse outcomes in fetus, includingh dyslipidemia, changes in liver physiology and female offspring fatty liver (126, 127). The evidence suggest exposure to MNPs *in utero* cause long term metabolic outcome in later life of exposed pups with some sex specific effects. Nevertheless, the MNPs used in the studies were manufactured and likely did not contain any of the myriad of chemical additives present in commercial plastic products. Some of these latter chemicals have been reported to promote programming of transgenerational adult-onset diseases (128, 129). Studies exposing both pregnant mice and rats to BPA, di(2-ethylhexyl) phthalate (DEHP), and dibutyl phthalate (DBP) observed obesity, puberal abnormalities, testis, and ovarian disease in F1 and F2 but more profound effects observed in F3 generation (128, 129), impacting fertility and reproduction for the future generation. These observations highlight that plastic chemicals alone cause programming through direct and indirect exposure to the subsequent generations and a combined effect of MNPs including chemicals may cause additional effects.

Additionally, exposure to PE (10-20μm) in parent mice and throughout offspring life, observed autistic-like traits including repetitive and compulsive behavior in offspring from post-weaning and into adulthood (130). Furthermore, exposure of pregnant mice to PS-MNPs led to NPs accumulation in the fetal thalamus, and the eight-week progenies observed anxiety-like behavior (99). Although the reports are limited, these observations are evidence that early exposure to MNPs can induce to long-term neurobiological disorders in offspring later in life. Microplastic exposure *in utero* and early life demonstrated short term effects in future progeny, however, investigations of the long-term effects are limited; therefore, future work is needed to identify long lasting fetal programming. Additionally, studies on the effects of a greater diversity of MNPs (e.g. polymer types, shapes, degree of weathering/UV treatment, etc) is needed as most studies only investigate the effects of PS-MNPs (131).

## 6 Remaining gaps in knowledge and high priority research areas

The available evidence on reproductive and developmental effects of MNPs exposures, although largely confined to studies of a single type of MNP (PS microspheres), suggests that significant impacts are possible. However, there remain considerable gaps in understanding that prevent a thorough assessment of whether current MNP exposures contribute to significant human infertility or disease. While humans are clearly ubiquitously exposed to diverse MNPs that probably infiltrate fetal tissues, limitations in current methods of measuring MNPs in various matrices (food, dust, tissue, etc) render any estimate of these exposures inaccurate, especially for particles < 1μm.

In addition, there are very few sources of well characterized, homogeneous preparations of microplastics available in sufficient quantity to study the potential hazards to reproduction. As such, most published toxicity studies have examined the effects of standardized polystyrene micro- or nano-sized plastics (PS-MNP). This contrasts with the great diversity of microplastic shapes, sizes, polymer matrices and their associated chemical content found among the MNP pollution in the environment. MNPs vary in size, shape, and chemical composition, it is possible that some types may be more harmful than others and the combined effect of particle and chemical may cause more damage than the polymer particle alone. Moreover, studies on rodents tend to use exposure rates in the 10s of millions of particles per kg body weight, which is estimated to be much higher than plausible, real-world exposures for humans or animals (131). Lower exposure rates of MNPs, that more plausibly reflect real world exposures, should be examined to better assess their true hazards. Furthermore, studies should consider the chemical additives present in the tested MNPs as these may contribute to the toxicity by being carried past the physiological defense mechanisms to vulnerable tissues. The extent to which these variables may influence toxicity remains unknown and research is needed to evaluate the potential health effects of different types of polymers and additives.

## 7 Conclusions

Abundant evidence makes clear that MNP particles contaminate the tissues of humans including within the womb. What is less clear is how much of these exposures are from nanoplastics and the extent to which these exposures influence fertility, fetal development and subsequent offspring health. Animal studies have revealed short-term impacts of MNP exposures but few have examined long-term, transgenerational impacts or if these impacts may occur at lower, environmentally-relevant rates of exposure. Future work is needed to further investigate long-term outcomes in individuals exposed to environmentally relevant MNPs *in utero*. Also, methods to characterize human MNP exposure – especially to nanoplastics - must be improved so that clinical and epidemiological studies can begin to assess real world impacts on human populations. This will also help to identify sources of MNPs exposure and develop mitigation strategies to limit risks. In addition, more accurate assessment of exposures to MNPs – quantitative and qualitative assessments – will improve the capacity to extrapolate harms predicted by other models of toxicity. Human plastic exposure will continue to grow rapidly based on the rate at which plastics are entering waste streams. Understanding the extent to which MNPs threaten the health of future generations will require considerable research effort.

## Author contributions

RZ: Writing – original draft. YC: Writing – review & editing. MW: Conceptualization, Supervision, Writing – original draft, Writing – review & editing. SB: Supervision, Writing – original draft, Writing – review & editing.
